# Supplementary material for: Identification of photoperiod-induced specific miRNAs in the adrenal glands of Sunite sheep (Ovis aries)
Source: Front Vet Sci. 2022 Jul 22;9:888207. doi: 10.3389/fvets.2022.888207 (PMC9354845; doi:10.3389/fvets.2022.888207)
Supplement: Supplementary file 1 [file Data_Sheet_1.ZIP › Supplementary file/miRDeep2 file.zip!/miRDeep2 file/Novel_Structure/SP42A2/Novel_92.pdf]

Provisional ID	:	NC_019464.2_17536
Score total	:	2
Score for star read(s)	:	-1.3
Score for read counts	:	0
Score for mfe	:	1.7
Score for randfold	:	1.6
Score for cons. seed	:	
Total read count	:	51
Mature read count	:	25
Loop read count	:	0
Star read count	:	26

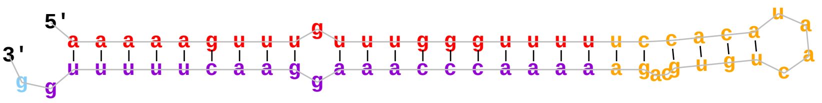

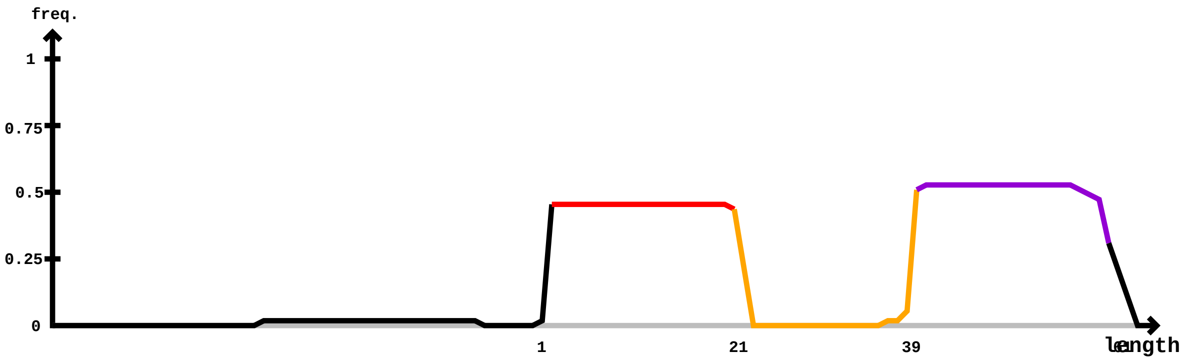

Mature Star

```
5'- caagaagauauaaauuugaauagaccugugcaguaucuauugguuuguccaaaaaguuuguuuggguuuuuccacauaacugugcagaaaaacccaaaggaacuuuuuggcca  -3'   obs
    caagaagauauaaauuugaauagaccugugcaguaucuauugguuuguccaaaaaguuuguuuggguuuuuccacauaacugugcagaaaaacccaaaggaacuuuuuggcca         exp
    ..................(((((((.(((.......))).)))))))(((((((((((.((((((((((((((((....))))..))))))))))))).)))))))))))...   reads   mm   sample
    ....................uagaccugugcaguaucuauugg......................................................................   1       0    seq
    .................................................caaaaaguuuguuuggguuC............................................   1       1    seq
    ..................................................aaaaaguuCguuuggguuuu...........................................   23      1    seq
    ..................................................aaaaaguuuguuuggguuuuu..........................................   1       0    seq
    .....................................................................................agaaaaacccaaaUgaacuuu.......   1       1    seq
    .......................................................................................aaaaacccaaaUgaacuu........   1       1    seq
    .......................................................................................aaaaacccaaaUgaacuuuu......   1       1    seq
    ........................................................................................aaaacccaaaUgaacuuuuu.....   9       1    seq
    ........................................................................................aaaacccaaaUgaacuuuuug....   16      1    seq
    .........................................................................................aaacccaaaUgaacuuuuuggc..   1       1    seq
```
